# Changes in Bone Mineral Density and Metabolic Parameters after Pulsatile Gonadorelin Treatment in Young Men with Hypogonadotropic Hypogonadism

**DOI:** 10.1155/2015/324524

**Published:** 2015-08-31

**Authors:** Chen-Xi Li, Song-Tao Tang, Qiu Zhang

**Affiliations:** Department of Endocrine and Metabolic Diseases, The First Affiliated Hospital of Anhui Medical University, Hefei 230032, China

## Abstract

To assess the prevalence of osteoporosis in young men with hypogonadotropic hypogonadism (HH) and to investigate the changes of BMD and metabolic parameters, a total of 22 young male patients with HH and 20 healthy controls were enrolled in the study. BMD, biochemical, and hormonal parameters were measured in two groups. Osteoporosis was more prevalent in HH patients (45.45%) than the control subjects (10.00%) (*P* < 0.001). The patients with HH had lower BMD in lumbar spine 2–4, femoral neck, and total hip (*P* < 0.001, for all) and higher fasting insulin (*P* = 0.001), HOMA-IR (*P* = 0.002), and SHBG (*P* < 0.001) compared to the controls. After 6 months of pulsatile gonadorelin treatment, BMI (*P* = 0.021) and BMD in lumbar spine 2–4, femoral neck, and total hip (*P* = 0.002, *P* = 0.003, and *P* = 0.003, resp.) increased dramatically and total cholesterol (*P* = 0.034), fasting insulin (*P* = 0.025), HOMA-IR (*P* = 0.021), and SHBG (*P* = 0.001) decreased significantly in HH patients. The study shows a higher prevalence of osteoporosis in young men with HH. Long-term pulsatile gonadorelin treatment indicates a positive effect on BMD and metabolic parameters of HH patients.

## 1. Introduction

Hypogonadotropic hypogonadism (HH) is a rare congenital or acquired disease characterized by the absent or inadequate secretion of gonadotropin-releasing hormone (GnRH) or gonadotropin [1]. Congenital HH is also called idiopathic hypogonadotropic hypogonadism (IHH), and it falls into two main types: Kallmann syndrome (KS) and normosmic IHH (nIHH). KS refers to the combination of HH and anosmia, while the olfaction of nIHH is normal. In male patients, HH can manifest as micropenis, small testis, azoospermia, or infertility. In addition, recent studies show that patients with HH are more likely to suffer from diabetes, lipid metabolic disorders, osteoporosis, and bone fracture due to their low testosterone levels [2, 3].

Hitherto, there are three main treatments for HH: testosterone preparation, gonadotropin including human chorionic gonadotropin (hCG) and human menopausal gonadotropin (hMG), and pulsatile gonadorelin [4–6]. Experimental data have demonstrated the favorable effects of testosterone on bone mineral density (BMD) in men with HH [7], and metabolic parameters have also been widely investigated by using both testosterone and gonadotropin [8]. Although research on GnRH therapy for HH has begun more than 30 years ago [9], the mechanisms of gonadal development and metabolic changes remain to be elucidated.

The aim of this study was to evaluate the prevalence of osteoporosis in young men with HH and to observe their BMD and metabolism parameters compared with the control group. Furthermore, changes of the above parameters were assessed in HH patients after 6 months of pulsatile gonadorelin.

## 2. Materials and Methods

### 2.1. Subjects

A retrospective study included 22 untreated young men with HH and 20 age- and sex-matched healthy controls who were recruited from the Department of Endocrine and Metabolic Diseases, The First Affiliated Hospital of Anhui Medical University, from December 2012 to February 2015. Inclusion criteria were (1) the failure to undergo a spontaneous puberty before the age of 18; (2) determination by a low testosterone level (*T* < 3.5 nmol/L) and gonadotropin within or below the normal range; (3) a normal karyotype (46, XY); (4) being with normal function of pituitary-thyroid axis, pituitary-adrenal axis, and pituitary-growth hormone axis; (5) receiving no treatment of testosterone or gonadotropin analogues before. Exclusion criteria were (1) suffering from traumatic brain injury or intracranial infection; (2) a family history of diabetes mellitus, pituitary tumors, familial hypercholesterolemia, hyperprolactinemia, or osteoporosis; (3) being with severe liver or kidney disease; (4) consanguineous marriage between parents or ancestors.

None of the patients and the controls had used drugs influencing bone, glucose, or lipid metabolism before. No bone fracture was seen in each individual. The study was approved by The Ethical Committee of The First Affiliated Hospital of Anhui Medical University, and an informed consent was obtained by all the participants, which was carried out in line with the Helsinki Declaration.

### 2.2. Measurement of BMI

Body height and weight of patients and healthy subjects were measured in the fasting state with their underwear. BMI was calculated by the ratio of weight to height squared (kg/m^2^).

### 2.3. Laboratory Measurements

Fasting blood samples were collected between 8 a.m. and 10 a.m. in all individuals. Follicle-stimulating hormone (FSH), luteinizing hormone (LH), and total testosterone (TT) were measured by chemiluminescence, using DXI800 (Beckman Coulter Inc., Miami, USA). Total cholesterol (TC), total triglyceride (TG), and high-density lipoprotein cholesterol (HDL-C) were measure by enzymatic colorimetric assay, using P800 (Roche Diagnostics Inc., Basel, Switzerland). Low-density lipoprotein cholesterol (LDL-C) was calculated by Friedewald's formula [10]. The fasting blood glucose (FBG) was determined by glucose oxidase method with BIOSEN C-line (EKF Inc., Hamburg, Germany). Fasting insulin and 25(OH)-Vitamin D were performed by chemiluminescence, using ADVITAL Centaur XP (Siemens Healthcare Diagnostics Inc., Dublin, Ireland) and sex hormone-binding globulin (SHBG) was determined by Immulite 2000 assay.

Insulin sensitivity was evaluated by using the homeostasis model assessment of insulin resistance (HOMA-IR) formula: HOMA-IR = fasting blood glucose (mmol/L) × fasting insulin (*μ*IU/mL).

### 2.4. BMD Measurements

BMD was measured by dual energy X-ray absorptiometry (DEXA) (GE Medical System Lunar Madison, WI, USA) on lumbar spine (L_2__–__4_), femoral neck (FN), and total hip (TH). As the ages of all the patients and control subjects were less than 50 years, *Z*-score values were applied to diagnose osteoporosis in the two groups. The diagnosis of normal BMD referred to *Z*-score above −2SD, and *Z*-score below −2SD was diagnosed with osteopenia. Those BMD below −2.5SD with obvious secondary factors of osteoporosis such as hypogonadism could be defined as osteoporosis [11].

### 2.5. Treatment and Specific Procedures

A synthetic GnRH, gonadorelin, was manufactured by Anhui Fengyuan Pharmaceutical Co., Ltd., and each ampule contained 100 *μ*g of gonadorelin. 600 *μ*g of gonadorelin was dissolved in 3 mL of normal saline, and then it was injected into a La Fenice microinfusion pump produced by Shanghai MicroPort Lifesciences Co., Ltd. Gonadorelin was infused into the patient's body via an embedded hypodermic needle linked to the microinfusion pump. Each dose of gonadorelin was 10 *μ*g within a 90 min pulse period. From 0:00 a.m., 160 *μ*g of gonadorelin in total was administered subcutaneously to patients with HH per day. The responses to pulsatile gonadorelin were monitored by clinical routine examination, and all the biochemical, hormonal, and densitometric variables were measured 6 months later.

### 2.6. Statistical Analysis

Statistical analysis was performed by using SPSS 16.0 package program (SPSS, Inc., Chicago, IL, USA). Kolmogorov-Smirnov test was used to determine the distribution characteristics of variables and Levene's test was used to evaluate the quality of variance. Data were expressed as mean values ± standard deviation (SD) and differences between parameters were analyzed by both independent sample and paired sample *t*-test. Nonparametric variables were compared with Chi-square test. *P* values <0.05 were considered statistically significant.

## 3. Results

### 3.1. Baseline Biochemical, Hormonal, and Densitometric Variables

The characteristics of the patients with HH and the control group are displayed in Table 1. There were no statistically significant differences in terms of age, BMI, and lipid profile including TC, TG, LDL-C, and HDL-C among the two groups. FSH, LH, and TT were significantly lower in patients with HH (*P* < 0.001, for all) than healthy controls, as expected. The young men with HH had higher fasting insulin (*P* = 0.001), HOMA-IR (*P* = 0.002), and SHBG (*P* < 0.001) in comparison with the control groups. However, FBG and 25(OH)-Vitamin D were not significantly different among the patients and the controls. For densitometric parameters, BMD was significantly lower in the patient group than the control group at lumbar spine 2–4, femoral neck, and total hip (*P* < 0.001, for all).

### 3.2. Comparisons for the Prevalence of Osteoporosis between the Two Groups

**Figure 1:**
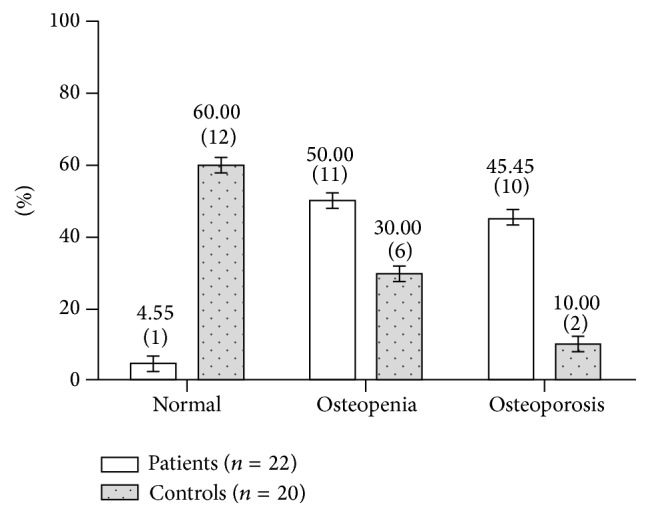
The prevalence of osteoporosis in patients and controls given as %(*n*).

According to the diagnosis criteria of osteoporosis above, individuals with osteoporosis were counted based on their *Z*-score and calculated to obtain the percentage of each group. There were 10 patients with osteoporosis accounting for 45.45% of the HH group, while only 2 persons were diagnosed with osteoporosis in the control subjects, constituting 10% of the group (Figure 1). The prevalence of osteoporosis between the two groups was significantly different (*χ* ^2^ = 16.053, *P* < 0.001).

### 3.3. Effects of Pulsatile Gonadorelin Treatment

Changes of biochemical, hormonal, and densitometric variables after 6 months of pulsatile gonadorelin treatment in patients with HH are shown in Table 2. Serum TC decreased, while BMI, FSH, LH, and TT increased after pulsatile gonadorelin treatment (*P* < 0.05, for all). However, no significant alterations were seen in aspect of TG, LDL-C, and HDL-C nor in FBG and 25(OH)-Vitamin D. The fasting insulin and HOMA-IR decreased significantly (*P* = 0.025 and *P* = 0.021, resp.) after the treatment. The increase of BMD was observed at lumbar spine 2–4, femoral neck, and total hip within the application of gonadorelin, and the difference was statistically significant (*P* < 0.01, for all).

## 4. Discussion

In this study, we report the higher prevalence of osteoporosis in young men with hypogonadotropic hypogonadism (HH) compared to the normal controls. Osteoporosis (OP) is characterized by a loss of bone mass as well as a deterioration of bone microstructure which leads to the increase of bone fragility and fracture risk [12]. Men osteoporosis is becoming an increasingly serious public health problem though it is often overlooked. An early study showed that almost one-third of hip fracture around the world occurred in men [13]. The primary or secondary hypogonadism is a major factor for the development of osteoporosis [14]. In men with HH and Klinefelter's syndrome and in women with Turner's syndrome, the decrease of cortical BMD was observed due to the adolescent gonadal steroid deficiency [7].

After 6 months of pulsatile gonadorelin treatment, BMD at lumbar spine 2–4, femoral neck, and total hip increased in the patient group. Several studies have demonstrated the role of testosterone in regulation of bone metabolism. It could directly combine with androgen receptors (ARs), or it is converted to dihydrotestosterone (DHT) with a high affinity by 5-alpha reductase before binding with ARs. Also, testosterone could act indirectly through oestrogen receptors (ERs) after peripheral aromatization. However, there is a paucity of data evaluating the correlation between GnRH and BMD. Recent studies showed that the immune system played an important regulative role in the development of osteoporosis [15]. In the peripheral blood of patients with OP, CD3+T lymphocytes, CD8+CD56+ lymphocytes, and CD4+/CD8+ ratio increased significantly. These functional lymphocytes could produce mass of inflammatory cytokines such as TNF-*α*, which promoted bone absorption and participated in the progress of OP. Interestingly, in a previous study, there was a decrease of CD4+/CD8+ ratio and immunoglobulin levels in male patients with IHH after gonadotropin treatment [16]. These studies indicate that GnRH may increase the secretion of gonadotropin to have an impact on bone metabolism through an indirect immunological way.

Vitamin D deficiency is also an important factor for the occurrence and development of OP. 25(OH)-Vitamin D (activated Vitamin D) contributes to the differentiation of progenitor cells and the production of osteocalcin and insulin-like growth factor-1 (IGF-1), enhances the activity of alkaline phosphatase, and ultimately accelerates the bone formation and mineralization. It was reported that 25(OH)-Vitamin D was significantly lower in hypogonadal men compared to healthy controls [17]. In our study, there was no significant difference between HH patients and controls. After the gonadorelin treatment, 25(OH)-Vitamin D increased in patients group, but it still showed no statistically significant difference. One possible reason may be the variation in sunlight exposure among the two groups. More 25(OH)-Vitamin D is absorbed via the skin as the irradiation time gets longer. The Vitamin D levels in the body fluctuate with the change of seasons [18], so patients who get admitted to the hospital in winter are more likely to have lower Vitamin D than in summer. More enrolled patients with consistent observation points are needed in the further study.

Sex hormone-binding globulin (SHBG) is a plasma glycoprotein produced by liver and binding with high affinity to sex steroids, and its level is regulated by multiple factors such as age, weight, sex steroids, or insulin [19]. The variation of SHBG levels is connected with a series of diseases (e.g., polycystic ovarian syndrome, insulin resistance, metabolic syndrome, and osteoporosis). Most studies have demonstrated that there is a negative relationship between SHBG levels and BMD. In a 3-year research on male osteoporosis, SHBG positively correlated with C-telopeptide of type I collagen (CTx) and free deoxypyridinoline (D-Pyr) which were bone absorption markers after adjustment for age, BMI, and sex steroids in both health controls and postmenopausal osteoporosis (PMOP) patients. A later study also showed the high SHBG levels in young men with HH [20]. Our study found the similar results in HH patients, and SHBG decreased with BMD increased after pulsatile gonadorelin treatment (*P* = 0.001). However, it remains to be elucidated whether SHBG could directly influence BMD or sex steroids indirectly.

Furthermore, there were lipid and glucose metabolic disturbances among patients with HH. Compared to the normal subjects, HH patients had higher fasting insulin and HOMA-IR, and lower profiles were observed after the application of gonadorelin. The results were consistent with relevant studies on the effect of testosterone in young men with HH [20, 21]. One possible hypothesis is that insulin resistance (IR) impairs the secretion of Leydig cell which produces testosterone in the body, and the low level of testosterone activates lipoprotein lipases to uptake more triglyceride (TG), and it will further deteriorate IR. The higher TG in the patient group compared with the control group may partly explain the mechanism of IR. In addition, studies in rodents have demonstrated that IR releases inflammatory cytokines and impairs GnRH neurons in the area of hypothalamus [22, 23]. Thus, we have reason to believe that the supplement of GnRH may have important influence on the reversal of inflammation caused by IR. Though a long-term testosterone therapy has shown the positive effect on hypogonadal men [24], the metabolic effects of either testosterone or gonadotropin are still controversial. Sonmez et al. [21] demonstrated the deterioration of TG and HDL-C after 3 weeks of testosterone esters. On the contrary, Schleich and Legros [25] reported that there were reduced TC, LDL-C, and HDL-C after physiological testosterone replacement. In a study integrating three medications including esterized testosterone (TE), human chorionic gonadotropin (hCG), and testosterone gel (TG) in patients with HH, the decrease of triglyceride was only observed in the hCG group while the parameter increased in TE and TG group [8]. Our study shows the positive effect of pulsatile gonadorelin treatment on triglyceride without changes of other lipid variables. Though the metabolic effects of gonadorelin have not been fully investigated, there is not enough evidence about the unfavorable impact of gonadorelin on cardiovascular diseases. However, the metabolic effects of each treatment in HH patients should still be highly valued.

## 5. Conclusions

This study has several limitations as follows: The lower incidence of HH restricts the recruited number of young male patients with HH. In addition, bone turnover markers and biopsy were not conducted due to budget constraints and the unwillingness of subjects. Finally, other treatments like gonadotropin or testosterone were not included to compare the difference of the curative efficacy in HH patients.

In conclusion, this study shows that there is a higher prevalence of osteoporosis among young men with HH accompanied with metabolic disturbance. And pulsatile gonadorelin treatment suggests a favorable effect on bone mineral density, insulin resistance, and lipid metabolism. Much attention should be paid to assess the risk of bone fracture in HH patients due to the low levels of bone mass brought by hypogonadism. Besides, the mechanism of gonadorelin on metabolic parameters is still unclear, so large-scale and multicenter observations are required in the follow-up studies to help patients with HH to choose the most suitable and effective treatment method.

## Figures and Tables

**Table 1:** Biochemical, hormonal, and densitometric parameters in patients and controls.

	Patients	Controls	*P*
	(*n* = 22)	(*n* = 20)	
Age (year)	21.15 ± 1.96	21.70 ± 1.35	0.462
BMI (kg/m^2^)	22.87 ± 4.06	21.50 ± 2.43	0.354
FSH (mIU/mL)	1.30 ± 1.04	5.24 ± 2.64	<0.001
LH (mIU/mL)	0.81 ± 0.68	4.50 ± 2.10	<0.001
TT (nmol/L)	0.78 ± 0.42	19.80 ± 5.21	<0.001
TC (mmol/L)	4.71 ± 1.43	3.96 ± 0.50	0.146
TG (mmol/L)	1.57 ± 1.13	0.88 ± 0.44	0.098
LDL-C (mmol/L)	2.51 ± 0.94	2.22 ± 0.54	0.394
HDL-C (mmol/L)	1.46 ± 0.35	1.44 ± 0.19	0.835
FBG (mmol/L)	5.50 ± 0.52	5.49 ± 0.33	0.970
Insulin (*μ*IU/mL)	21.27 ± 7.68	7.95 ± 2.36	0.001
HOMA-IR	5.31 ± 2.27	1.96 ± 0.66	0.002
25(OH)-Vitamin D (ng/mL)	15.49 ± 4.65	15.62 ± 5.52	0.955
SHBG (nmol/L)	45.46 ± 14.00	22.30 ± 5.12	<0.001
L_2__–__4_ (g/cm^2^)	0.905 ± 0.153	1.191 ± 0.085	<0.001
FN (g/cm^2^)	0.802 ± 0.111	1.051 ± 0.055	<0.001
TH (g/cm^2^)	0.838 ± 0.123	1.116 ± 0.069	<0.001

BMI: body mass index; FSH: follicle-stimulating hormone; LH: luteinizing hormone; T: testosterone; TC: total cholesterol; TG: total triglyceride; LDL-C: low-density lipoprotein cholesterol; HDL-C: high-density lipoprotein cholesterol; FBG: fasting blood glucose; HOMA-IR: homeostasis model assessment of insulin resistance; SHBG: sex hormone-binding globulin; L_2__–__4_: lumbar spine 2–4; FN: femoral neck; TH: total hip.

**Table 2:** Changes of biochemical, hormonal, and densitometric parameters in patients after 6 months of pulsatile gonadorelin treatment.

	Baseline	6 mon.	*P*
	(*n* = 22)	(*n* = 22)	
BMI (kg/m^2^)	22.87 ± 4.06	24.6 ± 3.64	0.021
FSH (mIU/mL)	1.30 ± 1.04	5.14 ± 3.64	0.008
LH (mIU/mL)	0.81 ± 0.68	4.97 ± 3.50	0.002
TT (nmol/L)	0.78 ± 0.42	10.86 ± 7.68	0.002
TC (mmol/L)	4.63 ± 1.97	4.41 ± 1.81	0.034
TG (mmol/L)	1.61 ± 1.12	1.59 ± 0.84	0.980
LDL-C (mmol/L)	2.51 ± 0.94	2.68 ± 1.41	0.151
HDL-C (mmol/L)	1.46 ± 0.35	1.74 ± 0.71	0.956
FBG (mmol/L)	5.50 ± 0.52	5.58 ± 0.49	0.888
Insulin (*μ*IU/mL)	22.38 ± 7.41	15.84 ± 5.15	0.025
HOMA-IR	5.31 ± 2.28	3.48 ± 1.77	0.021
25(OH)-Vitamin D (ng/mL)	15.49 ± 4.65	17.76 ± 5.92	0.498
SHBG (nmol/L)	45.46 ± 14.00	16.42 ± 8.95	0.001
L_2__–__4_ (g/cm^2^)	0.905 ± 0.153	1.017 ± 0.143	0.002
FN (g/cm^2^)	0.802 ± 0.111	0.923 ± 0.101	0.003
TH (g/cm^2^)	0.838 ± 0.123	0.920 ± 0.117	0.003

BMI: body mass index; FSH: follicle-stimulating hormone; LH: luteinizing hormone; T: testosterone; TC: total cholesterol; TG: total triglyceride; LDL-C: low-density lipoprotein cholesterol; HDL-C: high-density lipoprotein cholesterol; FBG: fasting blood glucose; HOMA-IR: homeostasis model assessment of insulin resistance; SHBG: sex hormone-binding globulin; L_2__–__4_: lumbar spine 2–4; FN: femoral neck; TH: total hip.
